# Therapeutic Potential of Noble Nanoparticles for Wound Repair

**DOI:** 10.5195/cajgh.2014.172

**Published:** 2014-12-12

**Authors:** Timur Saliyev, Gulsim Kulsharova, Alma Akhmetova, Talgat Nurgozhin, Sergey Mikhalovsky

**Affiliations:** 1 Center for Life Sciences, Nazarbayev University, Kazakhstan; 2 School of Engineering, Nazarbayev University, Kazakhstan; 3 School of Pharmacy and Biomedical Sciences, University of Brighton, UK

**Keywords:** wound management, cryogel, nanoparticles, silver, gold

## Abstract

**Introduction:**

Nanoparticles made of noble metals, such as gold and silver, have a great potential to be effectively employed for wound management. The nano-size of such particles provides an opportunity to enlarge the contacting area, which results in more effective anti-bacterial action and faster wound repair. It must be noted that the shape of noble nanoparticles might play a crucial role in the manifestation of their anti-microbial properties. The modern state of technology allows fabrication of the nanoparticles with the desired shape and physical properties. In order to provide efficacy and close contact with the wound, the noble nanoparticles can be incorporated into a special matrix made of a cryogel (based on polymethyl methacrylate). This combination might serve as a foundation for developing completely new types of wound dressing.

**Materials and methods:**

We have developed a few methods for synthesizing gold and silver nanoparticles of different shapes and sizes. After fabrication of metallic nanoparticles, they were characterized by using Tunneling Electron Microscopy (TEM) and Malvern Zetasizer system in order to determine the average population size and consistency. The silver nanoparticles was synthesized using sodium borohydride reduction of silver nitrate. The synthesis of gold nanoparticles was conducted by using the Turkevich method.

**Results:**

We have developed a synthetic cryogel based on polyacrylamide (by cryogelation reaction) at several temperatures. At the second step, we developed a method for conjugating fabricated gold and silver nanoparticles to the surface (or pores) of cryogel through covalent bonds so they can provide antibacterial action within the wound. By following the developed protocol, we were able to obtain an approximate cryogel layer (1 cm thickness) with embedded gold and silver nanoparticles. This conjugate was analyzed and confirmed using Scanning Electron Microscopy (SEM) and TEM.

**Discussion:**

The obtained results indicate the feasibility of the fabrication of a novel type of wound dressing. At the next step, we are planning to elucidate the bio-compatibility of the combination of cryogel and nanoparticles. Moreover, anti-bacterial properties of this new type of wound dressing will be analyzed.

